# Blood parameters and whole-blood transcriptomics associated with immune-related adverse events in metastatic renal cell carcinoma during nivolumab plus ipilimumab

**DOI:** 10.1038/s41598-026-46960-6

**Published:** 2026-04-23

**Authors:** Satoka Kinase, Yoshiyuki Nagumo, Bunpei Isoda, Hiromichi Sakurai, Reo Takahashi, Shuhei Suzuki, Akane Yamaguchi, Ryota Yanagihashi, Kozaburo Tanuma, Satoshi Nitta, Masanobu Shiga, Kosuke Kojo, Atsushi Ikeda, Takashi Kawahara, Akio Hoshi, Shuya Kandori, Bryan J. Mathis, Hiroyuki Nishiyama

**Affiliations:** 1 Department of Urology, University of Tsukuba, 1-1-1 Tennodai, Tsukuba, Ibaraki 305-8575 Japan; 2 Tsukuba Clinical Research & Development Organization (T-CReDO), University of Tsukuba, Tsukuba, Ibaraki 305-8576 Japan; 3 Department of Cardiovascular Surgery, Faculty of Medicine, University of Tsukuba, 1-1-1, Tennodai, Tsukuba, Ibaraki 305-8575 Japan; 4 Center for Cyber Medicine Research, University of Tsukuba, Ibaraki, Japan

**Keywords:** Immune checkpoint inhibitors, Immune-related adverse events, Biomarkers, Metastatic renal carcinoma, Gene expression profiling, Cancer, Computational biology and bioinformatics, Immunology

## Abstract

Pretreatment immune profiles associated with immune-related adverse events (irAEs) may inform individualized management strategies, including enhanced monitoring and early toxicity intervention, in patients with metastatic renal cell carcinoma (mRCC) treated with nivolumab plus ipilimumab. In this cohort (n = 51), we analyzed pretreatment peripheral blood parameters and whole-blood transcriptomic profiles. Higher lymphocyte and monocyte counts (odds ratios [ORs] 14.36 and 9.90, respectively) were significantly associated with an increased risk of irAEs, whereas higher neutrophil counts and C-reactive protein levels (ORs 0.08 and 0.27, respectively) were associated with a decreased risk. To characterize immune pathways associated with irAE development, we performed whole-blood transcriptomic analyses. Gene set enrichment analysis revealed upregulation of lymphocyte- and humoral immunity-related pathways and downregulation of neutrophil- and inflammatory-related pathways in patients who developed irAEs. CIBERSORTx demonstrated reduced neutrophil fractions and increased proportions of CD8⁺ T cells and activated memory CD4⁺ T cells, indicating an adaptive immune-dominant profile. Exploratory analysis identified five candidate genes (ANAPC1, CDK4, MCM6, GRAP2, and BST2) that may contribute to the immune features associated with irAE development. Collectively, these findings suggest that irAE development is associated with a distinct systemic immune profile characterized by enhanced lymphocyte-related and reduced neutrophil-related immune activity.

**Supplementary Information:**

The online version contains supplementary material available at 10.1038/s41598-026-46960-6.

## Introduction

Based on long-term follow-up results, the immune checkpoint inhibitor (ICI) nivolumab plus CTLA-4 inhibitor ipilimumab (Nivo + Ipi) combination has demonstrated superior overall survival (OS) and durable response benefits compared to protein kinase inhibitor sunitinib for advanced renal cell carcinoma (mRCC)^1^. However, immune-related adverse events (irAEs) remain a major concern since, in the CheckMate 214 trial, irAEs of any grade occurred in 80% of patients and 35% required high-dose glucocorticoids for severe events^2^. Although the incidence varies, Nivo + Ipi is consistently associated with higher rates of serious irAEs than nivolumab monotherapy, often leading to hospitalization and treatment interruption^2^. In this context, identifying molecular differences underlying irAEs and characterizing blood-based features associated with irAE development in mRCC treated with Nivo + Ipi are essential for enabling proactive toxicity management. Such approaches may support individualized management strategies, including enhanced monitoring and early intervention, to maximize treatment continuation while minimizing severe irAEs.

Previous studies had attempted to identify blood- based markers associated with irAE severity and clinical outcomes, including peripheral blood parameters, cytokines/chemokines, immunoglobulins^3,4^. Notably, peripheral blood count parameters (e.g., absolute lymphocyte, absolute monocyte, absolute eosinophil, and platelet counts) have been of persistent interest among clinicians and researchers as they are a straightforward, objective way to determine the probability of experiencing irAEs^5,6^. However, relatively few studies have examined the underlying immunological mechanisms that explain observed associations between these parameters and irAE development.

Whole-blood transcriptomic profiling may offer insights into these mechanisms, as it serves as a useful snapshot of immune activity by detailing interactions between immune cells circulating within blood and peripheral tissues. These cells may experience physiological conditions separate from the main tumor and have differing transcriptomes^7^. In particular, blood-based approaches have the advantage of being minimally invasive and suitable for longitudinal monitoring of immune-related changes^3,8,9^. Several studies have used whole-blood transcriptomic profiling to identify the pathway-level transcriptomic changes and potential biomarkers for distinguishing infections, characterizing autoimmune diseases, and predicting treatment responses in cancer^10–13^. However, in patients with mRCC, very few studies have investigated peripheral blood parameters associated with Nivo + Ipi and none, to our best knowledge, has evaluated pathway-level transcriptomic changes and immune features using whole-blood RNA transcriptomic profiling^14,15^.

In this study, we aimed to characterize the immunological and molecular features underlying peripheral blood parameters associated with irAE development in patients with mRCC treated with Nivo + Ipi, using integrated analyses of pretreatment blood parameters and whole-blood transcriptomic profiles.

## Materials and methods

### Patients and sample collection

For our prospective study, we collected whole blood samples from 51 mRCC patients using PAXgene Blood RNA tubes (PreAnalytix) before the administration of Nivo + Ipi at the University of Tsukuba Hospital between December 2016 and July 2023. All patients provided informed, written consent for the present study and the protocol was approved by the University of Tsukuba Hospital Institutional Review Board (#H28-104). Clinical data were obtained from hospital charts. All irAEs were graded by CTCAE (v 5.0) and dichotomized as irAE (−) = (grades 0–1) versus irAE (+) = (grades 2–4) because grades ≥ 2 typically require intervention and treatment interruption or hospitalization^7,16^.

### RNA isolation and RNA sequencing (RNA-seq)

Total RNA from whole-blood samples was isolated using a PAXgene Blood RNA kit (PreAnalytix) according to the manufacturer’s instructions. Globin RNA was removed from total RNA using a GLOBINclear-Human Kit (Thermo Fisher Scientific). For RNA sequencing, 500 ng total RNA were rRNA depleted using an NEBNext rRNA Depletion Kit (New England Biolabs, Hitchin, UK) according to the manufacturer’s instructions. Library preparation was performed using an NEBNext Ultra-Directional RNA Library Prep Kit (New England Biolabs). Libraries were validated via Bioanalyzer (Agilent Technologies, Santa Clara, CA, USA) to determine size distribution and concentration. Validated libraries were then sequenced at Tsukuba i-Laboratory LLP (Tsukuba, Japan) on a NextSeq500 (Illumina, San Diego, CA, USA) with the paired-end, 36-base read option.

### Differential gene expression analysis

We used the R package DESeq2 to identify differentially expressed genes (DEGs) between irAE (+) and irAE (−). Raw gene expression levels were normalized in DESeq2 and artifactual/low-expression genes were filtered. The threshold for defining significant DEGs was set to *p* < 0.05 and an absolute log2 (fold change) > 1.0 between the comparison groups. A procedure for false discovery rate adjustment (FDR) was applied; thus, an FDR-adjusted *p*-value < 0.05 was considered statistically significant.

### Gene set enrichment analysis

Gene Set Enrichment Analysis (GSEA) was performed using the fgsea package (version 1.32.4) in R with gene sets from the Molecular Signatures Database (MSigDB v7.5) accessed through the msigdbr package. Prior to GSEA analysis, gene ranking was performed by log2 (fold change) × − log10 (*p*-value). C2 canonical pathway gene sets from KEGG, Reactome, WikiPathways databases, and Hallmark gene sets (*p* < 0.05;FDR < 0.25) were selected for leading edge analysis. Gene sets containing human names were excluded to focus on established biological pathways. A normalized enrichment score (NES) was calculated to account for differences in gene set sizes, enabling cross-pathway comparisons. The analysis involved 10,000 gene set permutations with gene sets restricted to 10–500 genes.To ensure complete reproducibility, parallel processing was disabled (nproc = 1), random seed was fixed, and exact *p*-value calculation was enforced (eps = 0).

### Leading-edge analysis

Leading-edge analysis was performed on significantly enriched pathways (*p* < 0.05; FDR < 0.25) to identify core genes driving the enrichment signal^17^. Gene frequency across pathways was calculated to determine core contributors. An adaptive selection strategy was implemented in that genes appearing in ≥ 3 pathways were selected when 15–100 such genes existed. We used recurrence in ≥ 3 enriched pathways to reduce dependence on any single gene-set definition, suppress pathway-specific noise, prioritize genes contributing consistently across biological processes, and improve the robustness and interpretability of the panel.

### Digital cytometry analysis

To investigate the association gene expression and the cell component, we used CIBERSORTx with the LM22 signature matrix (relative mode, batch correction B-mode, quantile normalization disabled, 1000 permutations)^18^.

### Exploratory gene-signature analysis using penalized logistic regression

To deal with the small sample size and many features (*p* > *n*; 81 genes vs 51 samples), we adopted penalized logistic regression models using glmnet^19^ with the following settings: Ridge (α = 0), Elastic Net (α = 0.25, 0.5, 0.75), and Lasso (α = 1)^19–21^. Within each fit, the regularization parameter λ was chosen by the internal cross-validation routine of cv.glmnet using area under the curve (AUC) as the performance measure^22^. To address class imbalance, class weights were applied to both positive and negative classes^22^.

Our modeling followed a bootstrap-enhanced, penalized regression approach described by Abram and colleagues^20^. We used nested, stratified cross-validation (outer fivefold for n ≥ 50, otherwise threefold) to prevent information leakage^23^. Within each outer training split, features were selected by a bootstrap-based QNT procedure (95% CI)^20^. Specifically, penalized logistic regression was repeatedly fitted on bootstrap resamples to assess the stability of gene-level associations. For each gene, the empirical 95% confidence interval of the coefficient distribution was calculated. Genes were classified as QNT-positive when this 95% confidence interval did not include zero, indicating a stable direction of association across bootstrap resamples. To address class imbalance, a class-weighted glmnet model was then refit on the selected genes and evaluated on the held-out fold. Class weights were assigned inversely proportional to class prevalence within each training split.

For the final gene panel, we fixed the best α from the outer- cross-validated AUC, re-ran QNT on the full dataset, and ranked QNT-positive genes by mean bootstrap coefficients. Parsimonious size was chosen using the 1-SE rule from repeated cross-validation across nested panels^24^. Outer-cross-validated predictions were concatenated to compute ROC-AUC with 95% confidence intervals (CIs) by DeLong’s method (pROC) for performance^25^.

### Multivariable logistic regression model

After the penalized regression procedure, we fitted a standard multivariable logistic regression model using the selected candidate genes to estimate predicted probabilities and ROC characteristics of the candidate genes, rather than to serve as an independent validation model. Model fitting used maximum likelihood via the rms package (lrm) . Discrimination was accomplished via AUC (pROC) with 2000-bootstrap CIs; predicted probabilities were compared between irAE (+) and irAE ( −) by Wilcoxon’s rank-sum test.

### Statistical analysis

Categorical variables were compared using the Pearson χ^2^ test. Continuous variables were compared between groups using the Wilcoxon rank-sum test. To explore peripheral blood factors associated with irAEs, uni- and multivariate logistic regression analyses were used. For clinical interpretability, continuous peripheral blood variables were dichotomized using cutoff values determined by ROC curve analysis with the Youden index. Relative lymphocyte count, monocyte-to-lymphocyte ratio (MLR), and neutrophil-to-lymphocyte ratio (NLR) were excluded because of anticipated collinearity. All analyses were conducted in R (version 4.4.3 ).

## Results

### Patient characteristics and irAE profiles

**Fig. 1:**
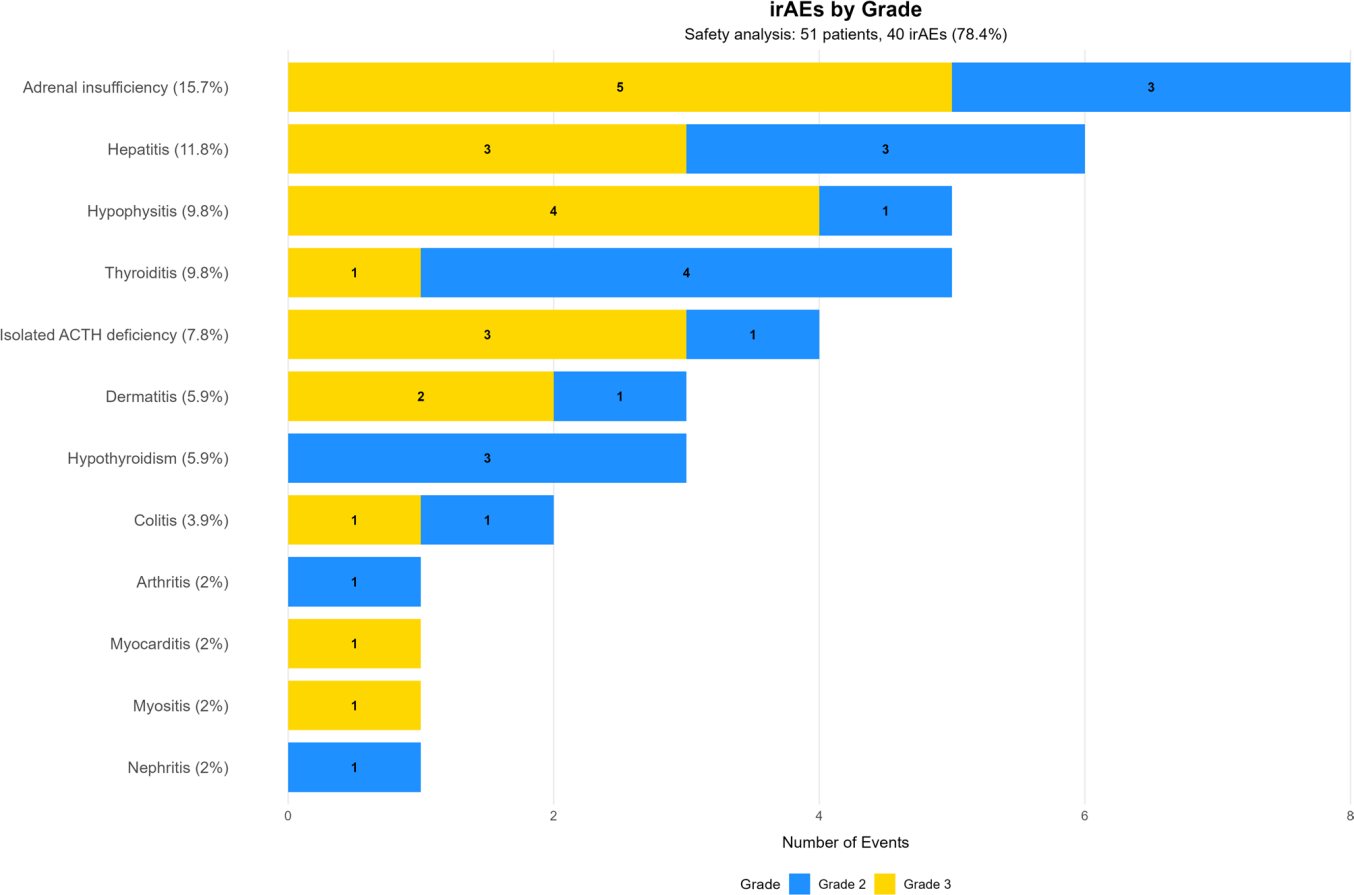
Distribution of irAEs by grade. Distribution of immune-related adverse events (irAEs) by CTCAE grade in the 51 patients. Bars indicate the number of events for each irAE; colors denote grade (≥ 2), and percentages in parentheses represent the proportion of patients who experienced each event.

Patient characteristics are summarized in Table 1 and median follow-up time was 18.8 months (range 2.9–77). The mean age of the cohort was 68 years (range 48–82) and the majority were male (80.4%). According to the International Metastatic RCC Database Consortium (IMDC) risk model, 2 patients (3.9%) were categorized as favorable risk, 38 (74.5%) as intermediate risk, and 11 (21.6%) as poor risk. Among baseline variables, only Eastern Cooperative Oncology Group (ECOG) performance status differed significantly between patients with and without irAEs, with the irAE (+) group showing poorer performance status (*p* = 0.008). During treatment, 29 patients (56.8%) experienced at least one irAE, including 10 patients who developed multiple events, resulting in a total of 40 irAEs observed. The median time to onset of the first irAE was 9 weeks (IQR, 7–12.1) and 16 patients (31.4%) discontinued treatment due to irAEs. The most frequent events were adrenal insufficiency (8 events, 15.7%), hepatitis (6 events, 11.8%), and hypophysitis (5 events, 9.8%) (Fig. 1).

**Table 1:** Baseline characteristics of patients.

Characteristic	irAE (−)	irAE (+)	*P*-value
Patients enrolled, n	29	22	
Age, years			
Median (range)	67 (48–75)	71 (50–82)	0.063
Gender, n (%)			0.483
Male	22 (75.9%)	19 (86.4%)	
Female	7 (24.1%)	3 (13.6%)	
ECOG performance status, n (%)			0.008
0	28 (96.6%)	15 (68.2%)	
1	0 (0.0%)	4 (18.2%)	
2	1 (3.4%)	3 (13.6%)	
IMDC risk score, n (%)			0.347
Favorable	0 (0.0%)	2 (6.9%)	
Intermediate	16 (72.7%)	22 (75.9%)	
Poor	6 (27.3%)	5 (17.2%)	
Histology, n (%)			0.447
Clear cell	26 (89.7%)	18 (81.8%)	
Non-clear cell	3(10.3%)	4(18.2%)	
Metastatic timing, n (%)			0.566
Synchronous	17 (58.6%)	15 (68.2%)	
Metachronous	12 (41.4%)	7 (31.8%)	
Sites of metastasis, n (%)			
Lung	17 (58.6%)	13 (59.1%)	1
Bone	7 (24.1%)	6 (27.3%)	1
Lymph node	12 (41.4%)	8 (36.4%)	0.778

### Baseline peripheral blood parameters associated with irAEs

We performed uni- and multivariate logistic regression analyses to reveal any associations between irAE occurrence and pre-treatment peripheral blood parameters. For each variable, the cutoff values, along with sensitivity and specificity are shown in Supplementary Table 1. In the univariate analysis, higher lymphocyte count was positively associated with irAE (+) whereas higher NLR, MLR, and CRP were negatively associated (Table 2). In the multivariate model, lymphocyte and monocyte counts remained positively associated with irAE (+), while neutrophil count and CRP were negatively associated. The wide confidence intervals may reflect the small sample size, sparse data structure, and reduced precision of parameter estimates. Taken together, these findings may indicate that baseline lymphocyte count may be an important factor associated with irAE occurrence.

**Table 2:** Univariate/multivariable binary logistic regression analysis.

Variables	Univariate OR	95% CI	*P*-value	Multivariate OR	95% CI	*P*-value
Lymphocyte count (μL; ≥ 2145)	6.11	1.40–43.06	0.03	14.36	1.45–292.31	0.043
Eosinophil count (μL; > 161)	2.49	0.78–8.62	0.132	1.11	0.18–6.59	0.906
Monocyte count (μL; > 409.5)	2.05	0.67–6.49	0.213	9.96	1.39–128.43	0.04
Neutrophil count (μL; > 3465)	0.41	0.08–1.67	0.239	0.08	0.01–0.62	0.03
CRP (mg/dL; > 0.11)	0.27	0.07–0.90	0.04	0.03	0.00–0.26	0.005
NLR (> 4)	0.21	0.05–0.71	0.016			
MLR (> 0.29)	0.21	0.05–0.71	0.016			

OR, odds ratio; CI, confidence interval; CRP, C-reactive protein; NLR, neutrophil-to-lymphocyte ratio; MLR, monocyte-to-lymphocyte ratio.

### Whole-blood transcriptomic and pathway enrichment analyses between irAE (+) and irAE (−)

**Fig. 2:**
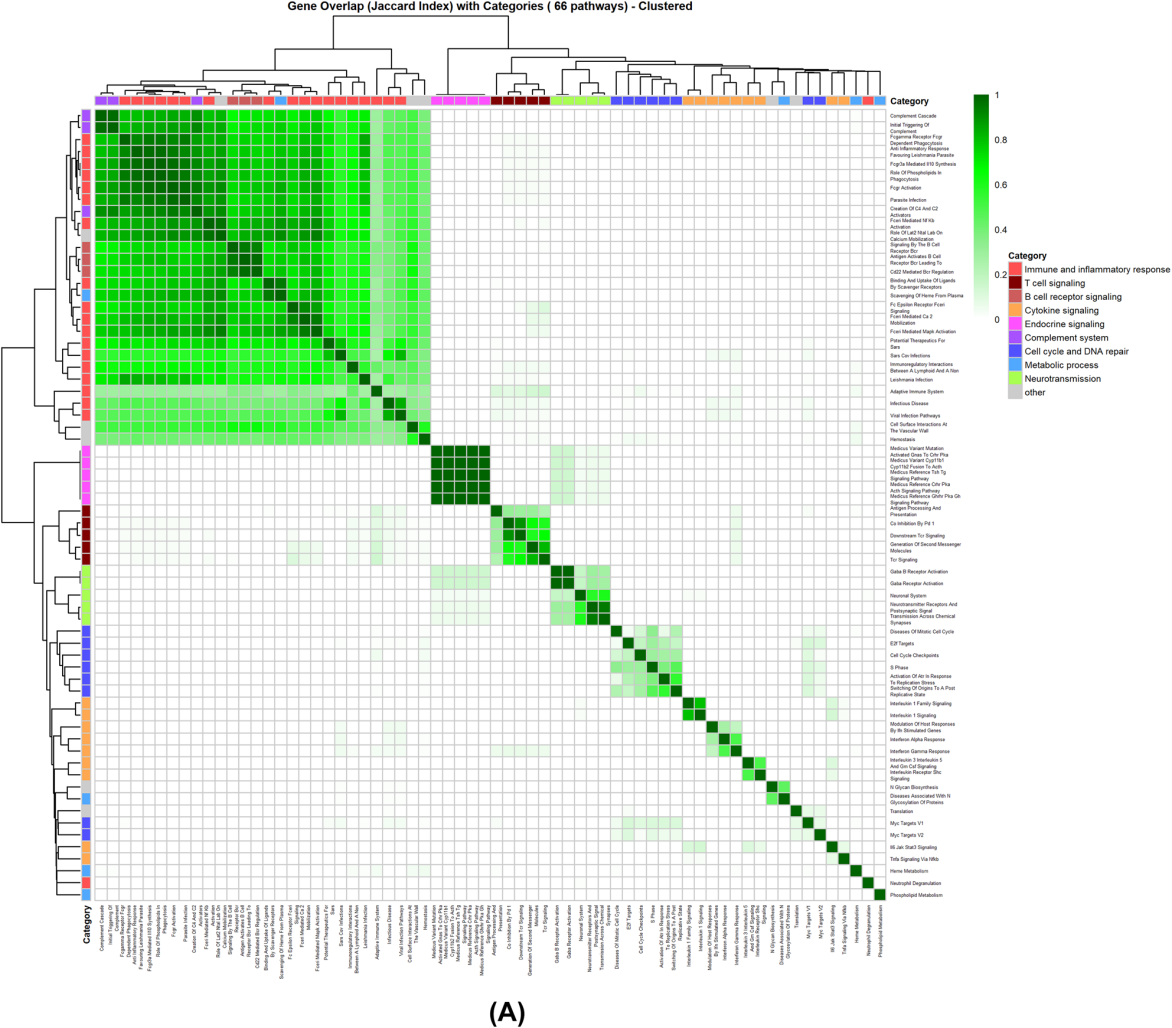

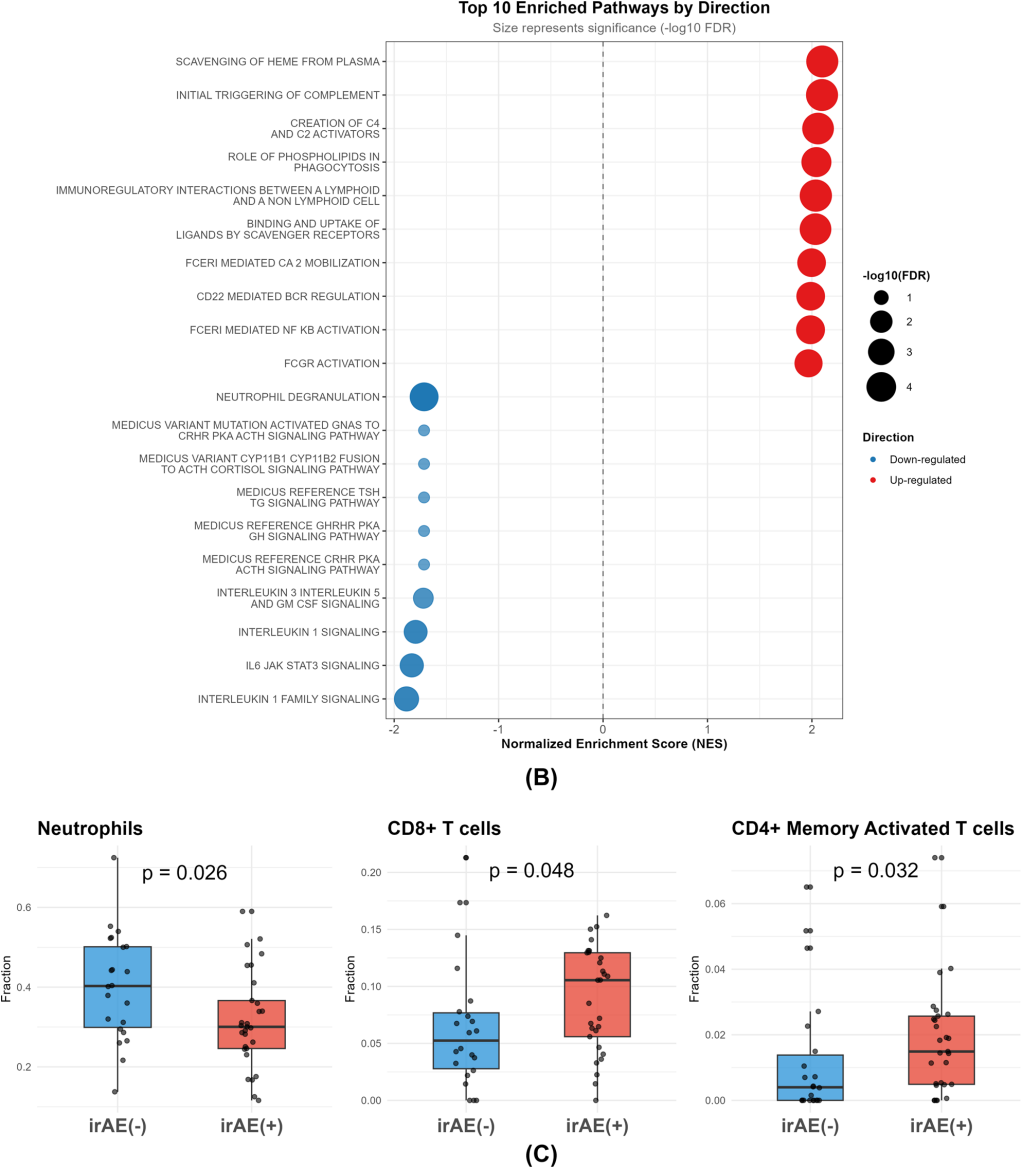
(**A**) A set-to-set heatmap was constructed from a pathway-by-pathway matrix using the Jaccard index of leading-edge genes Set-to-set heatmap of the 66 pathways significantly enriched in irAE (+) versus irAE ( −) patients (*p* < 0.05, false discovery rate (FDR) < 0.25). Each cell represents the Jaccard index of overlap between the leading-edge genes of a pair of pathways. The heatmap was generated in R (version 4.4.3) using the pheatmap package (version 1.0.13; https://cran.r-project.org/web/packages/pheatmap/index.html). (**B**) Dot plot of top 10 positive/negative, normalized enrichment score (NES) pathways. Dot plot showing the top 10 positively and top 10 negatively enriched pathways identified by gene set enrichment analysis (GSEA) comparing patients with and without immune-related adverse events (irAEs). The x-axis indicates the normalized enrichment score (NES), with each dot representing one pathway. Colors denote the direction of enrichment (red, up-regulated in irAE [+]; blue, down-regulated in irAE[−]), and dot size is proportional to -log10 of the false discovery rate (FDR), reflecting the significance of enrichment. (**C**) Box plots of significant cells between irAE (−) and irAE (+) using CIBERSORTx. Box plots show the estimated fractions of neutrophils, CD8⁺ T cells, and CD4⁺ memory-activated T cells in patients with irAE (+) and irAE (−). Cell fractions were inferred using CIBERSORTx with the LM22 signature matrix. Only immune-cell subsets with statistically significant differences between the two groups are shown. *P*-values were obtained using a two-sided Wilcoxon rank-sum test (*p* < 0.05).

To further characterize molecular differences associated with irAE development, we first explored transcriptomic differences between patients with and without irAEs by identifying 43 DEGs (|log2FC| ≥ 1.0, *p* < 0.05) (Supplementary Fig. 1A), although none remained significant after multiple testing correction. Pathway analysis suggested enrichment of immune-related pathways but the limited gene counts and potential overlaps warrant cautious interpretation (Supplementary Fig. 1B). Therefore, to identify transcriptomic changes at the pathway level, we applied GSEA to explore pathways and genes associated with irAEs. GSEA analysis revealed 66 significantly enriched pathways across KEGG (n = 7), Reactome (n = 51), and Hallmark (n = 8) databases (FDR<0.25, *p*<0.05) (Supplementary Table 2). A set-to-set heatmap demonstrated pathway overlap and functional clustering into nine categories (Figure 2A). The most dominant category was immune and inflammatory response, followed by cell cycle and DNA repair, then neurotransmission signaling, B-cell signaling, and endocrine signaling. Notably, among these immune-related pathways, B-cell and T-cell signaling were especially positively enriched, suggesting that pre-treatment upregulation of lymphocyte activity may predispose patients to irAE development. We then evaluated the statistical impact of each pathway (Figure 2B). Lymphocyte- and humoral immunity- related pathways, including BCR regulation, Fc receptor signaling, complement activation, were upregulated in patients with irAE (+). In contrast, neutrophil- and inflammatory-related pathways, such as neutrophil degranulation and IL-1, IL-6-JAK-STAT3, and IL-3/IL-5/GM-CSF signaling, were downregulated. These results suggest that immune activation patterns characterized by increased lymphocyte-related pathways and decreased neutrophil/inflammatory pathways may be associated with the development of irAEs.

Next, to investigate the immune cell profiles of blood samples, we applied digital cytometry using CIBERSORTx with the LM22 signature matrix (Supplementary Table 3)^26^. As shown in Fig. 2C, the irAE (+) group exhibited a reduction in neutrophil fraction (*p* = 0.026) and increases in CD8^+^ T cells (*p* = 0.048) and activated memory CD4^+^ T cells (*p* = 0.032). These findings indicate that irAE development is associated with an adaptive immune-dominant profile characterized by increased lymphocyte activity and reduced neutrophil predominance in line with the result from GSEA. Taken together, these results suggest that patients who already exhibit an active lymphocyte-dominant immune profile in pre-treatment whole blood are more likely to develop irAEs during Nivo + Ipi therapy.

### Exploratory gene signature analysis for developing irAEs

**Fig. 3:**
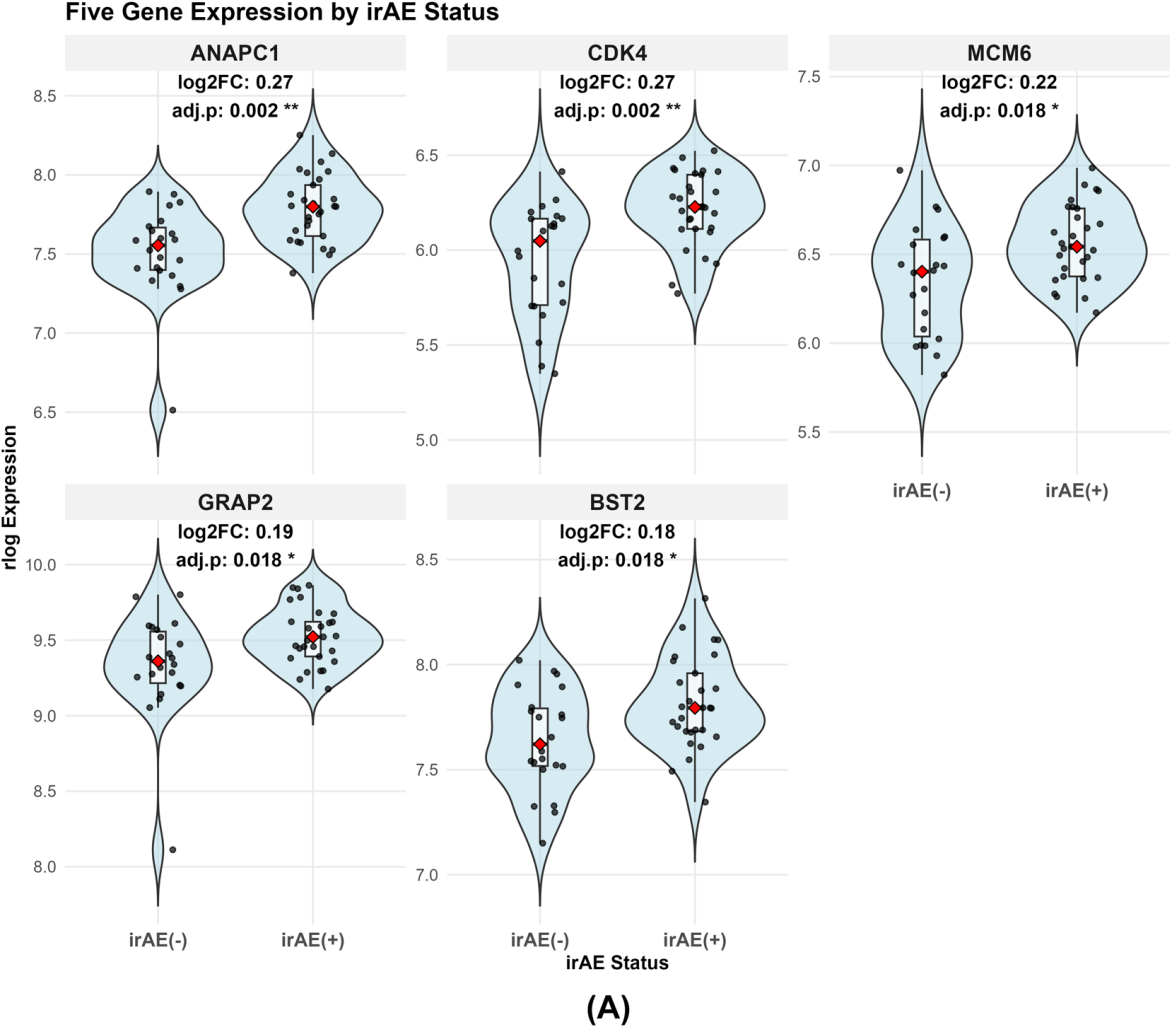

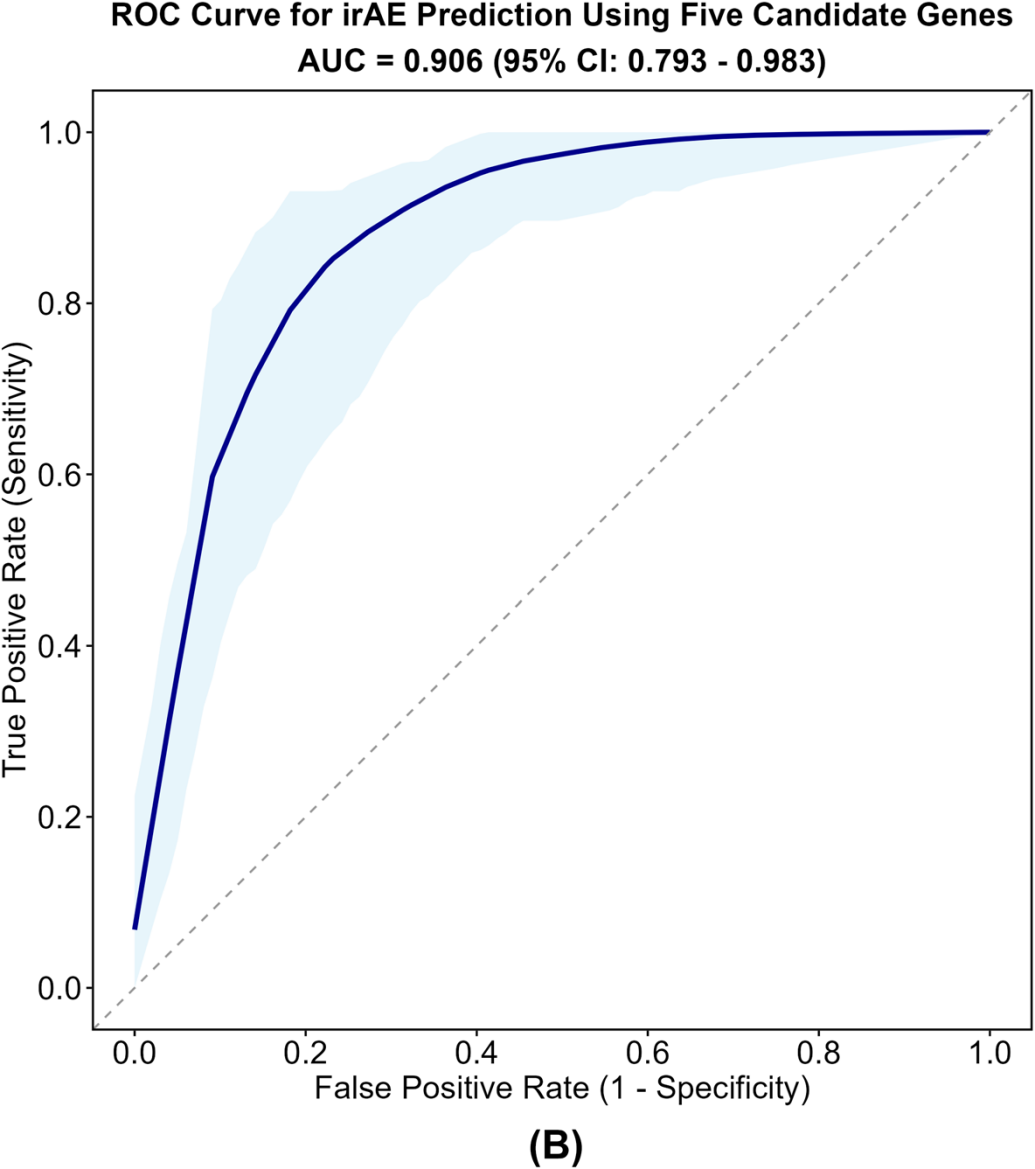
(**A**) Violin plots of expression levels of the five candidate genes. Violin plots showing baseline expression levels of the five candidate genes in patients with irAE (+) and irAE( −). Genes are ordered by log₂ fold change and Benjamin-Hochberg-adjusted *p*-values for irAE (+) versus irAE ( −); all five genes remained significant after multiple testing correction (adjusted *p*-value < 0.05). (**B**) ROC curve for irAE prediction by the five candidate genes. ROC curve of a multivariate logistic regression model based on the five-gene signature, yielding an AUC of 0.906 (95% CI, 0.793–0.983; *p* < 0.0001).

The following analysis was exploratory and hypothesis-generating. To explore the potential of whole-blood transcriptomic profile for predicting irAEs, we performed gene selection analysis as shown in the workflow (supplementary Fig. 2A and Table 4). Among five penalized regression models applied to 81 candidate genes from pathway analysis, ridge regression (α = 0) model identified five candidate genes (Supplementary Fig. 2B and Tables 5, 6). All five genes were more highly expressed in irAE (+) versus irAE (−) patients (all *p* values, adjusted < 0.05; Fig. 3A).

Next, we built multivariate logistic regression model as shown in Fig. 3B.The ROC curve of the five candidate genes model yielded an AUC of 0.906 (95% CI, 0.793–0.983; *p* < 0.0001). Using a probability threshold of 0.538, the model achieved a sensitivity of 93.1%, specificity of 77.3%, positive predictive value (PPV) of 84.4%, negative predictive value (NPV) of 89.5%, and an overall accuracy of 86.3%. These findings suggest that the five candidate genes have potential to serve as predictive biomarkers for irAEs, although the estimate should be interpreted cautiously because this exploratory analysis lacked external validation.

## Discussion

In this study, we demonstrated that irAE development was consistently associated with a pre-treatment immune profile characterized by increased lymphocyte counts and reduced neutrophil predominance. In addition, GSEA and digital cytometry analysis using CIBERSORTx further supported this lymphocyte-dominant state, highlighting T- and B-cell-related pathways as central to the pathogenesis of irAEs.

Previous studies have reported that lower NLR and MLR, together with higher lymphocyte counts in peripheral blood, are associated with irAEs^5,27,28^. We likewise identified baseline lymphocyte count as a significant and independent predictor of irAEs^28^ and interpret this as progression towards peripheral lymphocytosis, reflecting a state of T-cell activation that may predispose patients to immune-mediated toxicity^29^. Mechanistically, NLR would counter peripheral lymphocytosis through expansion of polymorphonuclear myeloid-derived suppressor cells (PMN-MDSCs) released from the bone marrow^30^ to suppress T lymphocytes; thus, a higher NLR could reflect increased MDSC expansion and a stronger immunosuppressive milieu in patients who did not develop irAEs^31^.

In addition, we observed that lower CRP at baseline was associated with irAEs as reported previously in RCC^32^. Because CRP expression is primarily induced by IL-6 via STAT3 activation acting together with hepatocyte transcription factors such as HNF-1α and C/EBP, lower CRP may indicate downregulated IL-6/STAT3 signaling at baseline, a finding also supported by our transcriptomic data^33–35^. It was also previously described that lower baseline levels of circulating IL-6 were associated with an increased risk of all grade irAEs in RCC^36^.

Consistent with these results, our GSEA showed lymphocyte- and humoral immunity-related pathways were upregulated, whereas neutrophil/inflammatory pathways, including the IL-6/JAK/STAT3 pathway, were downregulated in irAE (+) patients. Together with lower baseline CRP and previous reports linking low circulating IL-6 levels to a higher risk of irAEs, these findings suggest that patients who developed irAEs tend to have relatively low IL-6-driven systemic inflammation but a baseline state in which lymphocytes are more readily activated^36^. Collectively, these findings support the concept that a pre-activated, lymphocyte-dominant immune environment at baseline predisposes patients to irAEs, reflecting a heightened systemic immune readiness.

In an exploratory analysis, the five candidate genes we revealed as potential irAE biomarkers are comprised of one T-cell-related gene (GRAP2), three cell-cycling genes (CDK4, ANAPC1, MCM6), and one innate antiviral/type I interferon signaling gene (BST2). Our strategy of prioritizing genes that recurred across multiple enriched pathways was intended to improve robustness across pathway definitions. However, this approach may also introduce redundancy bias by favoring genes that are extensively represented in public pathway databases, potentially enriching for broadly recognized inflammatory pathways rather than irAE-specific mechanisms. Nonetheless, BST2 and GRAP2 are involved in pathways that have been implicated in irAE development, including antigen-specific T-cell responses and interferon signaling^37,38^, while CDK4 itself is responsible for T cell activation. ANAPC1 and MCM6 are necessary for cell cycle regulation and implicated in the progression of other cancers; they may also play a role in immune response^39–41^. Thus, we identified an exploratory five candidate genes related to T lymphocytes and cancer cell progression that may be associated with increased irAE risk.

Our study has several limitations. First, and most importantly, the sample size was modest (n = 51), and no independent external validation cohort was available. Therefore, the exploratory five-gene model, including its high apparent AUC of 0.906, remains at risk of overfitting and should be validated in a larger, independent cohort. Accordingly, this model should be interpreted as exploratory and hypothesis-generating. Second, dichotomization of continuous variables using the Youden index in multivariate logistic regression analyses may lead to loss of information and increase the risk of overfitting, resulting in overestimation of discriminative performance, particularly in small samples. Future studies with larger cohorts should assess these variables on a continuous scale to confirm our findings. Third, the use of whole-blood transcriptomic profiling may differ from tissue-specific expression patterns reported in previous studies. Finally, the cross-sectional design captures only baseline status, whereas longitudinal sampling could reveal changes over time associated with irAE development^42^. Despite these limitations, our findings provide a foundation for future studies to better understand the immune features and biological process that lead to irAE development.

From a practical perspective, peripheral blood parameters are less expensive, rapidly available, and routinely measured in daily clinical practice. In contrast, whole-blood RNA-seq remains more resource-intensive and requires specialized laboratory and bioinformatic workflows^43^. However, sequencing costs have declined substantially over time, and this shift might be key to embedding sequencing more deeply into routine clinical practice^44^. Future work may therefore focus on translating RNA-seq-derived signatures into simplified targeted assays that are more feasible for routine clinical use.

In conclusion, we identified distinct molecular differences between patients who developed irAE and those who did not, based on peripheral blood parameters and whole-blood transcriptomic profiles. Our findings suggest that irAE development is associated with a distinct systemic immune profile characterized by enhanced lymphocyte-related and reduced neutrophil-related immune activity.

## Supplementary Information

Below is the link to the electronic supplementary material.

Supplementary Material 1

Supplementary Material 2

Supplementary Material 3

Supplementary Material 4

Supplementary Material 5

Supplementary Material 6

Supplementary Material 7

## Data Availability

The processed RNA-seq gene expression data generated in this study have been deposited in the NCBI Gene Expression Omnibus (GEO) under accession number GSE319496. The raw sequencing data are not publicly available due to patient privacy concerns but may be made available from the corresponding author upon reasonable request.
