# Supplementary material for: Blood parameters and whole-blood transcriptomics associated with immune-related adverse events in metastatic renal cell carcinoma during nivolumab plus ipilimumab
Source: Sci Rep. 2026 Apr 23;16:18800. doi: 10.1038/s41598-026-46960-6 (PMC13272965; doi:10.1038/s41598-026-46960-6)
Supplement: Supplementary file 1 — Supplementary Material 1 [file 41598_2026_46960_MOESM1_ESM.docx]

*Supplementary Materials*

**Blood parameters and whole-blood transcriptomics associated with immune-related adverse events in metastatic renal cell carcinoma during nivolumab plus ipilimumab**

Satoka Kinase^1^, Yoshiyuki Nagumo^1^, Bunpei Isoda^1^, Hiromichi Sakurai^1^, Reo Takahashi^1^, Shuhei Suzuki^1^, Akane Yamaguchi^1^, Ryota Yanagihashi^1^, Kozaburo Tanuma^1^, Satoshi Nitta^1^, Masanobu Shiga^1^*, Kosuke Kojo^1,2^, Atsushi Ikeda^1^, Takashi Kawahara^1^, Akio Hoshi^1^, Shuya Kandori^1^, Bryan J. Mathis^3^, Hiroyuki Nishiyama^1,4^

^1^Department of Urology, University of Tsukuba, 1-1-1 Tennodai, Tsukuba, Ibaraki 305-8575, Japan

^2^Tsukuba Clinical Research & Development Organization(T-CReDO), University of Tsukuba, Tsukuba, Ibaraki 305-8576, Japan

^3^Department of Cardiovascular Surgery, Faculty of Medicine, University of Tsukuba, 1-1-1, Tennodai, Tsukuba, Ibaraki 305-8575, Japan

^4^Center for Cyber Medicine Research, University of Tsukuba, Ibaraki, Japan

*Corresponding author: Dr. Masanobu Shiga, Department of Urology, University of Tsukuba, 1-1-1 Tennodai, Tsukuba, Ibaraki 305-8575, Japan.

Tel.: +81-29-853-3223, Fax: +81-29-853-8854. Email: mshiga@md.tsukuba.ac.jp

**Supplementary Figure 1A.** Volcano Plot of Differential Gene Expression Between irAE (+) and irAE (−)


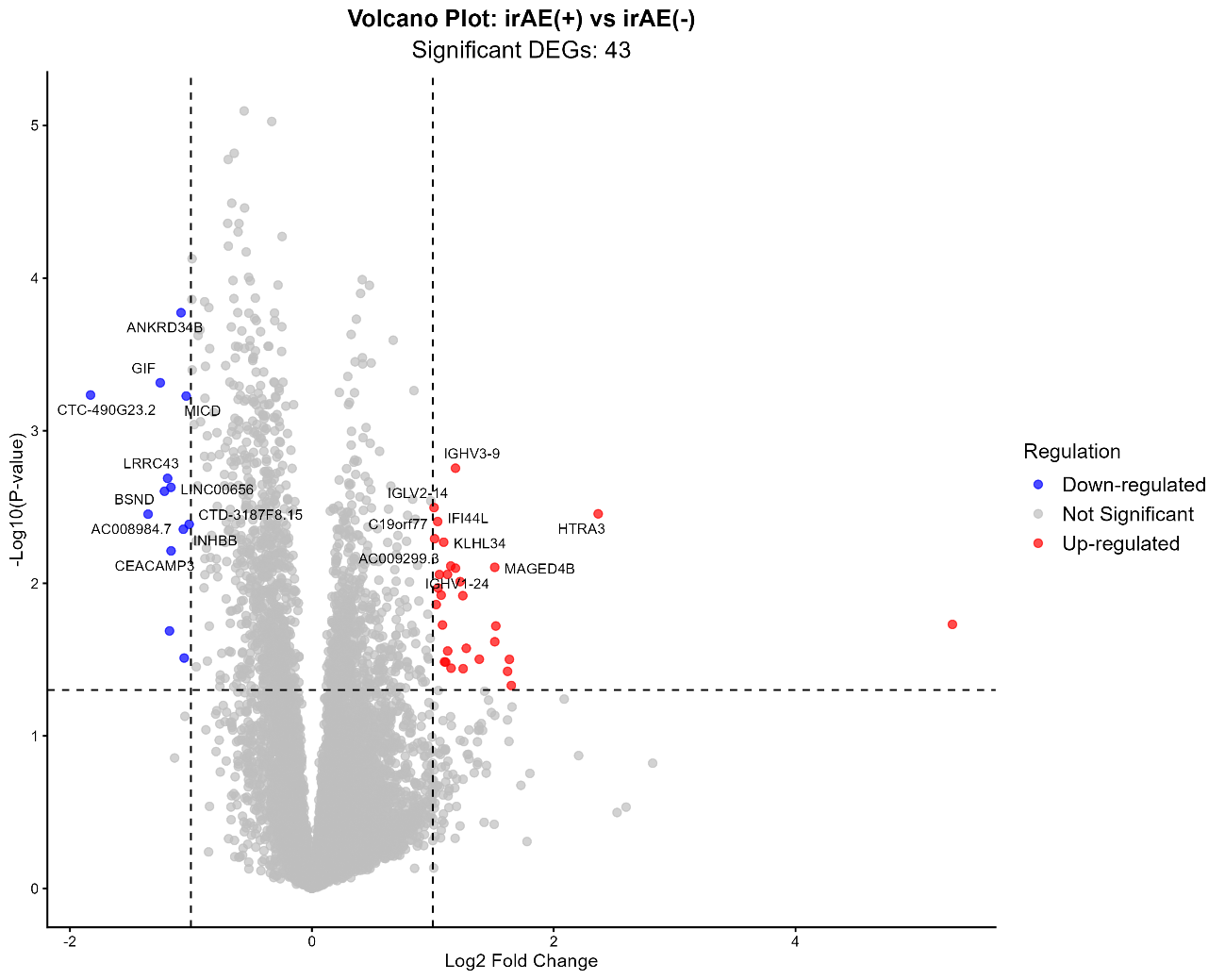


**Supplementary Figure 1B**. Dot plot of KEGG pathway


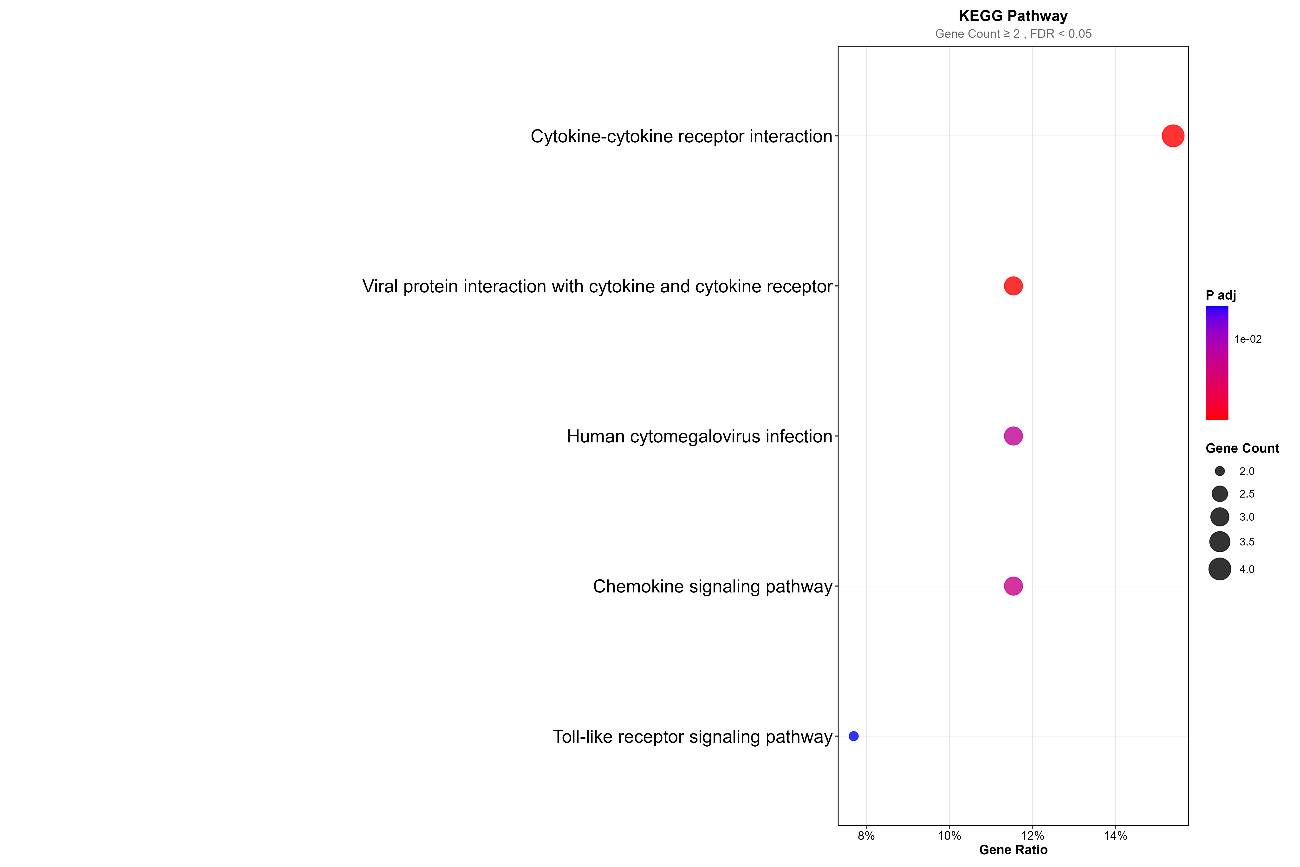


**Supplementary Figure 2A.** Analysis workflow of the present study

**
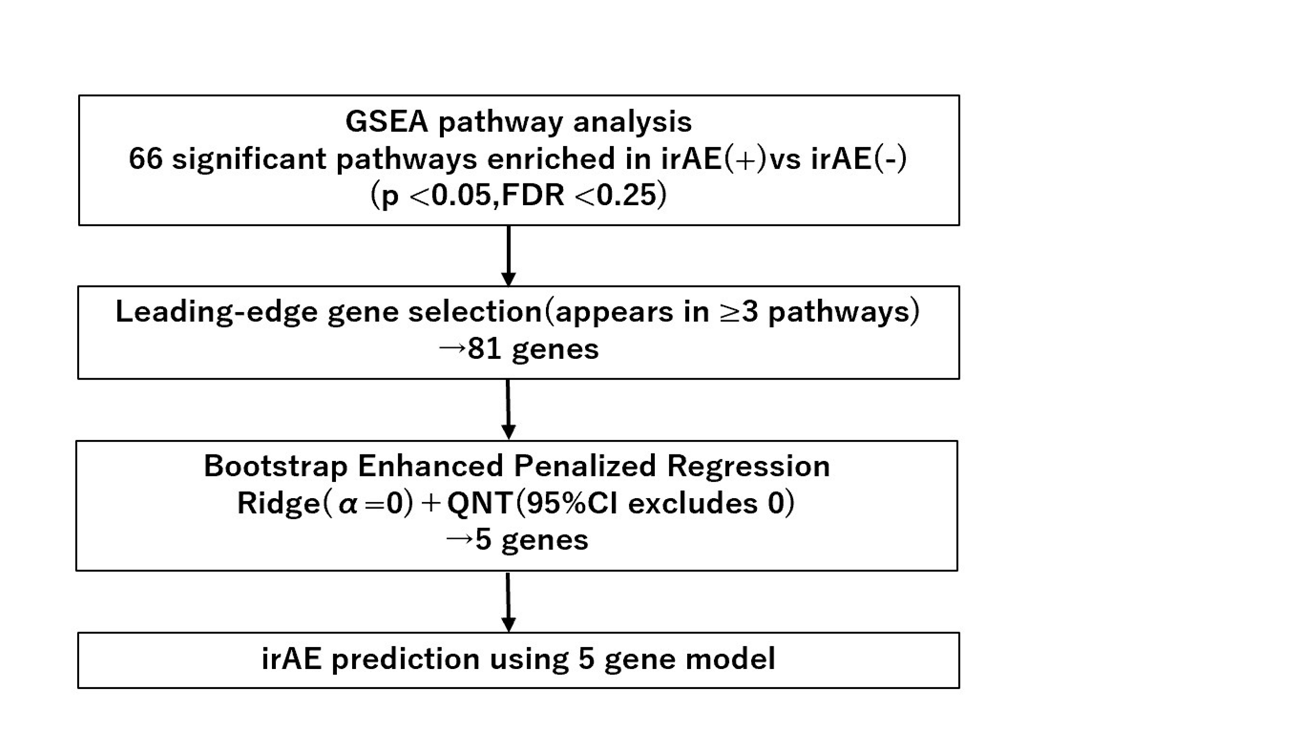
**

**Supplementary Figure 2B.** Outer cross-validated performance of the ridge logistic regression model for predicting irAEs.


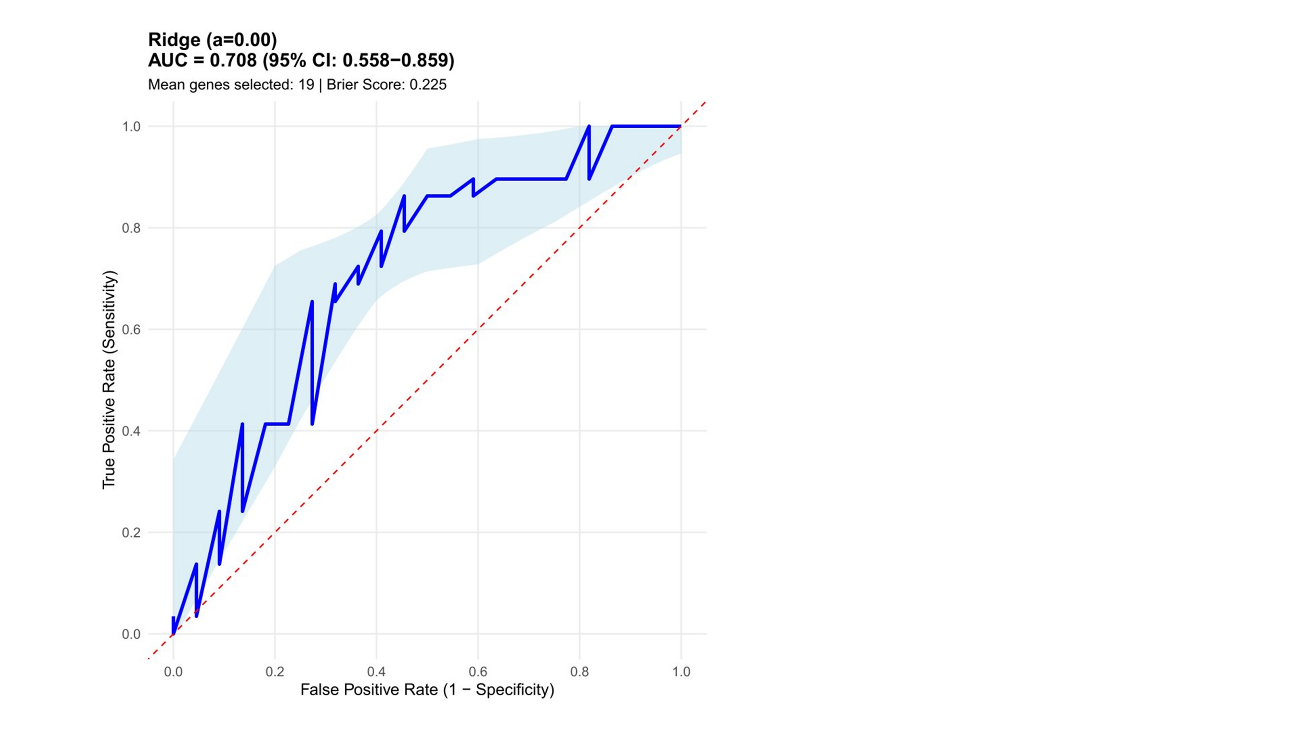


**Supplementary Table 1.** Receiver operating characteristic analysis of baseline peripheral-blood parameters for predicting irAEs

| Variables | Sensitivity | Specificity | AUC |
| --- | --- | --- | --- |
| NLR (>4) | 82.80% | 50.00% | 0.624 |
| MLR (>0.29) | 82.80% | 50.00% | 0.591 |
| Lymphocyte count (μL；≥2145) | 37.90% | 90.90% | 0.643 |
| Eosinophil count (μL；>161) | 48.30% | 72.70% | 0.534 |
| Monocyte count (μL；>409.5) | 58.60% | 59.10% | 0.505 |
| Neutrophil count (μL；>3465) | 27.60% | 86.40% | 0.528 |
| CRP (mg/dL；>0.11) | 51.70% | 77.30% | 0.618 |

**Supplementary Table 2.** Significantly enriched pathways identified by gene set enrichment analysis of baseline whole-blood transcriptomes. Full pathway list is provided as Supplementary Data 1.

| Pathway | Category | P-Values | P-Values, Adjusted | NES | Gene Set Size | Database |
| --- | --- | --- | --- | --- | --- | --- |
| E2F TARGETS | Cell cycle and DNA repair | 0.002 | 0.017 | 1.633 | 84 | Hallmark |
| HEME METABOLISM | Metabolic process | < 0.001 | 0.005 | 1.652 | 106 | Hallmark |
| IL6 JAK STAT3 SIGNALING | Cytokine signaling | < 0.001 | 0.004 | -1.831 | 48 | Hallmark |
| INTERFERON ALPHA RESPONSE | Cytokine signaling | < 0.001 | 0.001 | 1.909 | 49 | Hallmark |
| INTERFERON GAMMA RESPONSE | Cytokine signaling | < 0.001 | 0.005 | 1.672 | 94 | Hallmark |
| MYC TARGETS V1 | Cell cycle and DNA repair | 0.022 | 0.150 | 1.437 | 81 | Hallmark |
| MYC TARGETS V2 | Cell cycle and DNA repair | 0.034 | 0.201 | 1.496 | 20 | Hallmark |
| TNFA SIGNALING VIA NFKB | Cytokine signaling | 0.004 | 0.028 | -1.543 | 95 | Hallmark |
| ANTIGEN PROCESSING AND PRESENTATION | T cell signaling | 0.004 | 0.169 | 1.628 | 54 | KEGG |

**Supplementary Table 3.** Digital cytometry estimates of immune cell fractions in whole blood using CIBERSORTx

| CellType | SD  irAE (+) | Median  irAE (+) | SD  irAE (-) | Median  irAE (-) | Fold Change | P-Value | Significance |
| --- | --- | --- | --- | --- | --- | --- | --- |
| Neutrophils | 0.120 | 0.300 | 0.135 | 0.403 | 0.79 | 0.026 | * |
| T.cells.CD4.memory.activated | 0.018 | 0.015 | 0.019 | 0.004 | 1.539 | 0.033 | * |
| T.cells.CD8 | 0.046 | 0.106 | 0.056 | 0.053 | 1.395 | 0.048 | * |
| Mast.cells.resting | 0.012 | 0.016 | 0.014 | 0.022 | 0.774 | 0.140 | n.s. |
| Dendritic.cells.activated | 0.002 | 0.003 | 0.003 | 0.002 | 1.275 | 0.149 | n.s. |
| Monocytes | 0.077 | 0.281 | 0.0953 | 0.243 | 1.103 | 0.201 | n.s. |
| T.cells.gamma.delta | 0 | 0 | 0.0084 | 0 | 0 | 0.268 | n.s. |
| Macrophages.M1 | 0 | 0 | < 0.001 | 0 | 0 | 0.268 | n.s. |
| Macrophages.M2 | 0 | 0 | < 0.001 | 0 | 0 | 0.268 | n.s. |
| Dendritic.cells.resting | 0 | 0 | < 0.001 | 0 | 0 | 0.268 | n.s. |
| NK.cells.resting | 0.045 | 0.074 | 0.048 | 0.065 | 1.154 | 0.380 | n.s. |
| NK.cells.activated | 0.009 | 0 | 0.009 | 0 | 0.788 | 0.428 | n.s. |
| T.cells.CD4.naive | 0.036 | 0.084 | 0.037 | 0.082 | 1.099 | 0.540 | n.s. |
| Macrophages.M0 | 0.005 | 0 | 0.006 | 0 | 0.890 | 0.620 | n.s. |
| T.cells.CD4.memory.resting | 0.060 | 0.034 | 0.052 | 0.018 | 1.382 | 0.629 | n.s. |
| Plasma.cells | 0.007 | 0.010 | 0.007 | 0.006 | 1.103 | 0.641 | n.s. |
| B.cells.memory | 0.004 | 0 | 0.005 | 0 | 0.825 | 0.671 | n.s. |
| T.cells.regulatory..Tregs. | 0.020 | 0.006 | 0.016 | 0.006 | 1.153 | 0.691 | n.s. |
| Eosinophils | < 0.001 | 0 | 0.005 | 0 | 0.105 | 0.729 | n.s. |
| B.cells.naive | 0.031 | 0.027 | 0.027 | 0.027 | 1.029 | 1 | n.s. |
| T.cells.follicular.helper | 0 | 0 | 0 | 0 | NA | NA | NA |
| Mast.cells.activated | 0 | 0 | 0 | 0 | NA | NA | NA |

**Supplementary Table 4.** Leading-edge genes appearing in ≥3 pathways.

The full gene list is provided in Supplementary Data 2.

| Gene Name | Pathway Occurrence | Percentage (%) |
| --- | --- | --- |
| IGHV2-5 | 24 | 48 |
| IGHV2-70 | 24 | 48 |
| IGHV3-11 | 24 | 48 |
| IGHV3-13 | 24 | 48 |
| IGHV3-30 | 24 | 48 |
| IGHV3-53 | 24 | 48 |
| IGHV3-7 | 24 | 48 |
| IGHV4-34 | 24 | 48 |
| IGHV4-59 | 24 | 48 |
| IGKV1-33 | 24 | 48 |
| IGKV1D-12 | 24 | 48 |
| IGKV1D-39 | 24 | 48 |
| IGKV2-28 | 24 | 48 |
| IGKV2-30 | 24 | 48 |
| IGKV2D-28 | 24 | 48 |
| IGKV3-11 | 24 | 48 |
| IGKV3-15 | 24 | 48 |
| IGKV3-20 | 24 | 48 |
| IGKV4-1 | 24 | 48 |
| IGLC2 | 24 | 48 |
| IGLC3 | 24 | 48 |
| IGLV1-44 | 24 | 48 |
| IGLV1-47 | 24 | 48 |
| IGLV1-51 | 24 | 48 |
| IGLV2-11 | 24 | 48 |

**Supplementary Table 5.** Outer cross-validated performance of penalized logistic regression models for predicting irAEs

| Method | AUC | CI Lower | CI Upper | Mean AUC  (Across Folds) | SD AUC  (Across Folds) | Brier Score | Mean Number of Genes |
| --- | --- | --- | --- | --- | --- | --- | --- |
| Ridge  (α = 0) | 0.708 | 0.558 | 0.859 | 0.743 | 0.099 | 0.225 | 19.2 |
| ElasticNet  (α = 0.25) | 0.473 | 0.323 | 0.624 | 0.5 | 0 | 0.246 | 0 |
| ElasticNet  (α = 0.5) | 0.473 | 0.323 | 0.624 | 0.5 | 0 | 0.246 | 0 |
| ElasticNet  (α = 0.75) | 0.473 | 0.323 | 0.624 | 0.5 | 0 | 0.246 | 0 |
| Lasso  (α = 1) | 0.473 | 0.323 | 0.624 | 0.5 | 0 | 0.246 | 0 |

**Supplementary Table 6**. Performance of candidate gene panels derived from

bootstrap-enhanced penalized regression

| Number of Genes | AUC |
| --- | --- |
| 5 | 0.873 ± 0.004 |
| 6 | 0.855 ± 0.004 |
| 7 | 0.866 ± 0.005 |
| 8 | 0.869 ± 0.005 |
| 9 | 0.862 ± 0.004 |
| 12 | 0.862 ± 0.004 |
| 14 | 0.853 ± 0.004 |
| 16 | 0.845 ± 0.004 |
| 18 | 0.838 ± 0.004 |
| 20 | 0.838 ± 0.004 |
| 22 | 0.839 ± 0.004 |
| 23 | 0.837 ± 0.005 |

**Figure captions**

**Supplementary Figure 1A.** Volcano Plot of Differential Gene Expression Between irAE (+) and irAE (−).

Volcano plot showing differential gene expression between patients with irAE (+) and irAE (−). Red and blue dots indicate up- and down-regulated genes (|log₂ fold change| ≥ 1.0 and p < 0.05); 43 genes met these criteria. Only the top 20 genes are labeled for clarity.

**Supplementary Figure 1B**. Dot plot of KEGG pathway.

KEGG pathway enrichment analysis of the 43 differentially expressed genes. Pathways with FDR < 0.25 are displayed.

**Supplementary Figure 2A.** Analysis workflow of the present study

Baseline whole-blood gene expression profiles from patients treated with nivolumab plus ipilimumab (Nivo+Ipi) were analyzed to identify molecular differences between patients with and without immune-related adverse events (irAEs) using gene set enrichment analysis (GSEA). Leading-edge genes appearing in ≥3 enriched pathways were selected and further filtered by a quantile-based (QNT) procedure, then bootstrap-enhanced ridge regression models were constructed to derive a five-gene signature for irAE prediction.

**Supplementary Figure 2B.** Outer cross-validated performance of the ridge logistic regression model for predicting irAEs.

A penalized logistic regression model with a ridge penalty (α = 0) was trained on leading-edge genes using nested stratified cross-validation (outer 5-fold CV). Within each outer training split, genes were selected by a bootstrap-based quantile (QNT) procedure, and the class-weighted model was refit on the selected genes and evaluated on the held-out fold. The blue line shows the ROC curve based on all outer-fold predictions, and the shaded band represents ±1 standard deviation of sensitivity across outer folds at each false-positive rate. The cross-validated AUC of the ridge model was 0.708 (95% CI, 0.558-0.859), with a mean of 19 genes selected per outer fold and a Brier score of 0.225.
